# Rapid Separation and Detection of Drugs in Complex Biological Matrix Using TD-CDI Mass Spectrometer

**DOI:** 10.3390/bios14060271

**Published:** 2024-05-25

**Authors:** Wenyan Shi, Zi Ye, Qin Yang, Jianhua Zhou, Jiasi Wang, Xinming Huo

**Affiliations:** 1 School of Biomedical Engineering, Shenzhen Campus of Sun Yat-sen University, Shenzhen 518107, China; shiwy22@sz.tsinghua.edu.cn (W.S.); yezi28@mail2.sysu.edu.cn (Z.Y.); zhoujh33@mail.sysu.edu.cn (J.Z.); wangjs8@mail.sysu.edu.cn (J.W.); 2 Tsinghua Shenzhen International Graduate School, Tsinghua University, Shenzhen 518055, China; 3 Key Laboratory of Sensing Technology and Biomedical Instruments of Guangdong Province, School of Biomedical Engineering, Sun Yat-sen University, Guangzhou 510275, China

**Keywords:** thermal desorption, corona discharge ionization, drug analysis, analyte separation, mass spectrometry

## Abstract

The drug detection technology plays a pivotal role in the domains of pharmaceutical regulation and law enforcement. In this study, we introduce a method that combines thermal desorption corona discharge ionization (TD-CDI) with mass spectrometry for efficient drug detection. The TD-CDI module, characterized by its compact and simple design, enables the separation of analytes within seconds and real-time presentation of one or two analyte peaks on the mass spectrum most of the time, which reduces matrix interference and improves detection performance. Through experimental investigation, we studied the characteristics of TD-CDI for analyte separation and detection, even with the same mass number, and optimized the TD-CDI approach. TD-CDI-MS was employed for the rapid detection of drugs in various traditional medicine, food products, and human samples. Additionally, by utilizing TD-CDI for segmented hair direct analysis, it becomes possible to trace the drug usage cycle of individuals. This underscores the feasibility of the proposed analytical method within the realm of drug detection.

## 1. Introduction

The development of pharmaceutical technology has led to an increase in the variety, forms, and functions of drugs. However, the misuse of drugs such as opium, heroin, methamphetamine, and morphine can result in severe physical and mental dependence [1]. Drug abuse can cause damage to various organ systems, including the nervous system, cardiovascular system, and respiratory system [2]. This can result in symptoms such as muscle twitching, myocardial ischemia, myocardial infarction, and acute renal failure [3,4,5]. More seriously, drug abuse is responsible for thousands of deaths every year [6,7]. Many countries, including the United States and China, place great importance on drug supervision and require rapid detection technology for drugs and drug abusers.

Among various detection methods, mass spectrometry (MS) is widely used due to its high sensitivity, accuracy, and specificity. The combination of chromatography and mass spectrometry [8,9,10] can separate and detect analytes with high accuracy. However, the detection process is complicated and time-consuming, making it difficult to achieve rapid detection. In recent decades, mass spectrometry technology has rapidly developed. The emergence of ambient ionization mass spectrometry (AIMS) [11,12,13,14,15] enables direct sampling and ionization of analytes from their natural environment, thereby improving the on-site detection capabilities of mass spectrometers. Various AIMS techniques have been applied in the field of drug detection, including desorption electrospray ionization (DESI) [16], direct analysis in real time (DART) [17], paper spray ionization (PSI) [18], and low-temperature plasma ionization (LTP) [19], etc.

In addition to the ionization method, appropriate sampling techniques are also crucial in drug detection due to the diverse forms of samples [20,21,22] to be analyzed, including liquid samples such as beverages, sewage, blood, and urine, as well as solid samples such as capsules, tablets, and hair. Thermal desorption (TD) [23] is a sampling technique that can be used to separate and vaporize organic substances from matrices by heating. This method is suitable for complex samples in various forms, such as liquids and solids. It can also reduce the interference of the sample matrix on the substance being measured, as different substances can be desorbed at different times and temperatures [24]. The combination of TD and AIMS is an optional strategy to enhance the on-site drug detection capabilities of mass spectrometers. Up to now, ion sources, such as chemical ionization (CI) [25], electrospray ionization source (ESI) [26,27], ultraviolet photoionization (UVP) [28,29], and corona discharge ionization source (CDI) [30], have been coupled with TD devices to achieve drug detection and analysis. The CDI method employs a corona discharge generated by a DC high-voltage needle tip to ionize sample molecules in a gaseous medium. The advantage of this technology is that it requires no complex AC high-voltage control and can be operated under atmospheric pressure, making it suitable for use in field detection applications. However, in the process of detecting complex actual samples, the matrix often contain many interfering substances which will also be thermally desorbed. It will increase the complexity of mass spectrometry information and affect the effective identification of drug targets [31]. Therefore, most existing thermal desorption ionization technologies can only be used for the detection of some simple matrix samples, or it can only be effective when combined with high-resolution mass spectrometers. How to achieve the rapid detection of trace drugs in complex substrates, especially in biological samples, remains an important challenge.

In this work, a simple and convenient strategy for drug separation and detection using thermal desorption corona discharge ionization (TD-CDI) was proposed. The integrated thermal desorption room and hollow corona needle design enable effective desorption, separation, and ionization of mixed analytes in the time dimension, which simplifies the noise peak interference within a single spectrum. By optimizing the temperature and reagents, it was possible to effectively separate and identify substances with the same mass-to-charge ratio but different boiling points. And the feasibility of using the TD-CDI to detect drugs in milk tea, urine, capsules, and hair has also been confirmed. These studies are aimed at the rapid and efficient detection of drugs in complex samples.

## 2. Materials and Methods

### 2.1. Chemicals and Materials

Methanol, acetonitrile, formic acid, deionized water, and magnesium sulfate were purchased from Anpel Company (Shanghai, China). Dioxopromethazine hydrochloride (DPZ) was purchased from Aladdin Reagent (Shanghai, China). Gemcitabine was purchased from APExBIO Technology (Houston, TX, USA). Methamphetamine, tramadol, ketamine, and fentanyl were provided by Guangdong Nantian Institute of Forensic Science. Mimic urine was purchased from XingHeng Co., Ltd. (Dongguan, China). Milk tea was purchased from local shopping malls, and Anweiyang capsules were purchased from local pharmacies.

### 2.2. Instrumentation

**Figure 1:**
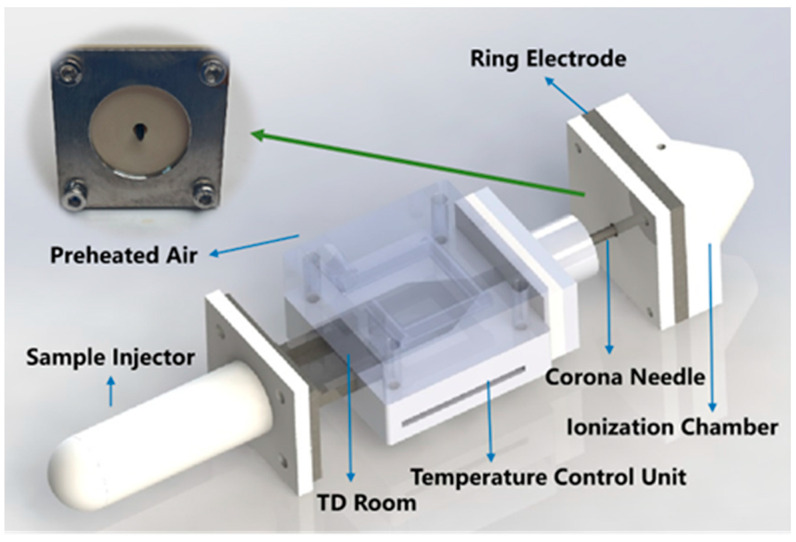
The structure drawing of the TD-CDI device.

As shown in Figure 1, the integrated TD-CDI device was optimized based on our previous studies [32,33]. It consists of a thermal desorption chamber and a corona discharge ionization chamber, both of which are tightly connected by a hollow corona discharge needle. The TD chamber is mainly composed of a hand-held sample injector, a temperature control unit, a TD room, and a preheated air purge system. The corona ionization source consisted of a hollow discharge needle with a high voltage of 5 kV and a grounded stainless steel ring electrode. The TD chamber is continuously purged with air at a flow rate of 1 L/min. And the temperature of thermal desorption can be fixed at room temperature to 250 °C as needed by the temperature control unit, with an accuracy of 1 °C. During the experiment, the analytes are desorbed after the injector with the sample is heated, and then transported into the CDI chamber under the action of purge gas and pressure difference to be ionized by the high-voltage corona discharge. The whole module is highly integrated, compact, and portable, which ensures that the analytes after desorption can be directly ionized and sent to MS analysis. All experiments were carried out on the LCQ-Fleet ion trap mass spectrometer (Thermo Fisher Scientific, Waltham, MA, USA). Unless otherwise specified, the mass spectrometry analysis employed the positive ion full scan mode, with the original sheath gas, auxiliary gas, and sweep gas deactivated. Additionally, the capillary temperature was set to 200 °C. And the parameters of the ion lens and detection system were automatically optimized by the LCQ Tune function.

## 3. Results and Discussion

### 3.1. Basic Separation Effect Verification

**Figure 2:**
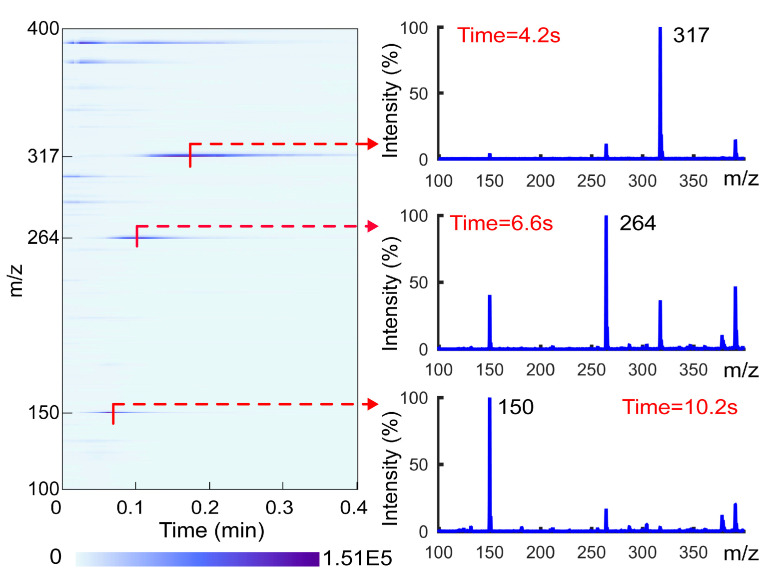
The ion map and characteristic spectra for a mixed solution of methamphetamine, tramadol, and dioxpromazine hydrochloride.

As mentioned above, most ion sources lack the ability to separate materials. This poses a challenge to material identification when detecting complex samples, as peaks from different substances will interfere with each other. The TD unit is capable of vaporizing and separating the analytes from the matrix. As the desorption energy required for different analytes varies, the desorption time for different analytes will also vary during the continuous heating process. This can simplify the mass spectrum, similar to the function of chromatography. To verify its separation function, a mixed standard solution of 1 μg/mL methamphetamine, tramadol, and dioxpromazine hydrochloride was prepared with methanol. A total of 10 μL of the solution was used for detection, and the desorption temperature was set to 240 °C. The ion map and spectra obtained after heating for 4.2 s, 6.6 s, and 10.2 s are shown in Figure 2. When heated for 4.2 s, the spectrum was dominated by the characteristic peak *m*/*z* 150 of methamphetamine; when heated for 6.6 s, it was dominated by the characteristic peak *m*/*z* 264 of tramadol; and when heated for 10.2 s, it was dominated by the characteristic peak *m*/*z* 317 of dioxpromazine hydrochloride. It is evident that, at a specific moment, there are only a few characteristic peaks of the target substance on the spectrum. This indicates that the use of TD-CDI can achieve a clear separation of different substances in the mixed sample in a short amount of time. TD-CDI is a potentially effective method to improve the simplicity and accuracy of mass spectrometry identification. In order to facilitate the subsequent description of the separation speed and separation results, the desorption time is defined as the time required for the detection sample to appear the strongest signal peak of the analyte.

### 3.2. Performance Optimization

**Figure 3:**
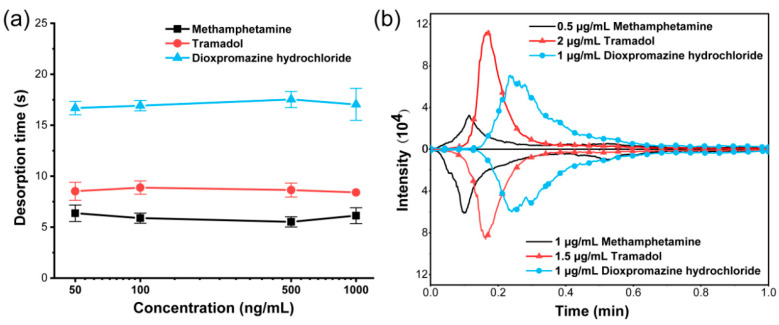
(**a**) The desorption time for different concentrations of methamphetamine, tramadol, and dioxpromazine hydrochloride in single standard solutions. (**b**) The extracted ion currents for different concentrations of methamphetamine, tramadol, and dioxpromazine hydrochloride mixed samples.

After verifying the feasibility of TD-CDI to separate substances, the performance of TD-CDI was optimized. The desorption separation depends mainly on the interaction between the sample and the TD module. For complex mixed samples, the concentration, components, and solvents of the sample may affect the energy absorption process or ionization process, thereby affecting the desorption time and material separation ability. In order to study the effect of different analyte concentrations on the material separation ability, methanol was used as the solvent to prepare single standard solutions of dioxpromazine hydrochloride, tramadol, and methamphetamine with concentrations of 50 ng/mL, 100 ng/mL, 500 ng/mL, and 1000 ng/mL, respectively. The desorption temperature was set at 200 °C, and 10 μL samples were used for detecting. The ion chromatograms of samples with various concentrations were observed in selected ion monitoring (SIM) mode, and the desorption time of the target was recorded. The desorption time obtained after taking the average value of multiple parallel experiments are shown in Figure 3a. It can be seen that the desorption time of different concentrations of dioxpromazine hydrochloride is about 17 s, the desorption time of different concentrations of tramadol is about 8.6 s, and the desorption time of different concentrations of methamphetamine is about 6 s. The results indicated that for samples containing only one single analyte, variations in concentration do not affect the desorption time within the specified range.

The impact of the analyte concentration in the mixed sample on the material separation performance also needs to be investigated. Two mixed samples were prepared using methanol as the solvent. The first one contained 0.5 μg/mL of methamphetamine, 2 μg/mL of tramadol, and 1 μg/mL of dioxetazine hydrochloride. The second one contained 1 μg/mL of methamphetamine, 1.5 μg/mL of tramadol, and 1 μg/mL of dioxetazine hydrochloride. The same conditions as the above experiments were set up, and the two mixed samples were detected using the TD-CDI. The extracted ion currents of the three analytes are shown in Figure 3b. Both mixed samples had a desorption time of about 6.3 s for methamphetamine, about 9.9 s for tramadol, and about 14.4 s for dioxpromazine hydrochloride. Then, we conducted a significance analysis for the desorption time data of different analytes at various concentrations obtained from Figure 3. It was found that regardless of whether it was mixed samples or single-labeled samples, there was no significant difference among different concentrations. However, there were extremely significant differences among the three analytes (*p* < 0.001). The results indicate that the concentration of the analyte has little impact on the desorption time, and the differences in desorption time can be utilized to separate different analytes. In addition, it can also be seen from this experimental result that the peak intensity of the analyte increases with the concentration, indicating the quantitative capability of the TD-CDI to some extent. Therefore, when using TD-CDI to detect real samples, different analytes can be stably separated even though the sample concentration is unknown. Operators can focus on the characteristic peaks of one or two analytes at a certain moment, so as to reduce the complexity of substance identification and improve the efficiency of substance identification.

**Figure 4:**
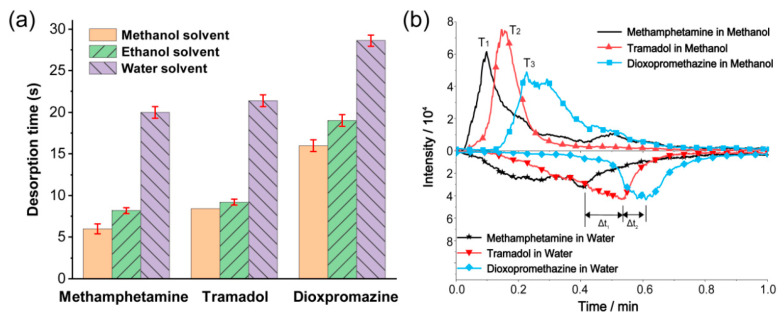
(**a**) The desorption time for different solvents of methamphetamine, tramadol, and dioxpromazine hydrochloride in single standard solutions. (**b**) The extracted ion currents of methamphetamine, tramadol, and dioxpromazine hydrochloride in mixed solutions with methanol and water as solvents.

The sample contains a high proportion of solvents, which could significantly affect the desorption separation ability. In order to study the influence of solvents on the detection performance of the TD-CDI, single standard solutions of 1 μg/mL dioxpromazine hydrochloride, 1 μg/mL tramadol, and 1 μg/mL methamphetamine were prepared using methanol, acetonitrile, and deionized water as solvents, respectively. The desorption temperature was set to 200 °C, and 10 μL samples were used for detection each time. The desorption time for each sample was obtained as shown in Figure 4a. The data indicate that the type of solvent used has an obvious impact on the desorption time. Specifically, when water is used as the solvent, the desorption time is longest, whereas when methanol is used, it is shortest. A possible reason is that the solvent is the primary component in the sample. As the sample enters the TD room, the analyte vaporizes mainly by absorbing heat though the solvent. This process may be related to the structure of the solvent molecules and intermolecular forces, which will affect the heat capacity and boiling point of the sample, thereby affecting the vaporization and endothermic process, resulting in differences in the desorption time.

Similarly, the effect of solvents on the desorption performance for mixed samples was also investigated. Mixed solutions containing 1 μg/mL dioxypromazine hydrochloride, 1.5 μg/mL tramadol, and 1 μg/mL methamphetamine were prepared using methanol and deionized water as solvents, respectively. The samples were detected by the TD-CDI under the same conditions as described above. The extracted ion currents are shown in Figure 4b. The results indicate that although using water as a solvent slightly increases the desorption time difference (Δt1 and Δt2), it also leads to longer desorption time, slower desorption speed, and weakened peak intensity of the analyte signal. Furthermore, when the signal peak of one substance is detected, the relative signal intensity of other substances is not low, leading to subpar separation performance. However, the separation performed well when methanol was used as the solvent, and the performance of acetonitrile as the solvent was comparable to that of methanol. Therefore, when conducting on-site detection that requires the use of liquid solvents to prepare samples, it is recommended to use organic solvents with low boiling points, such as methanol and acetonitrile. This will ensure high detection efficiency and good separation performance.

**Figure 5:**
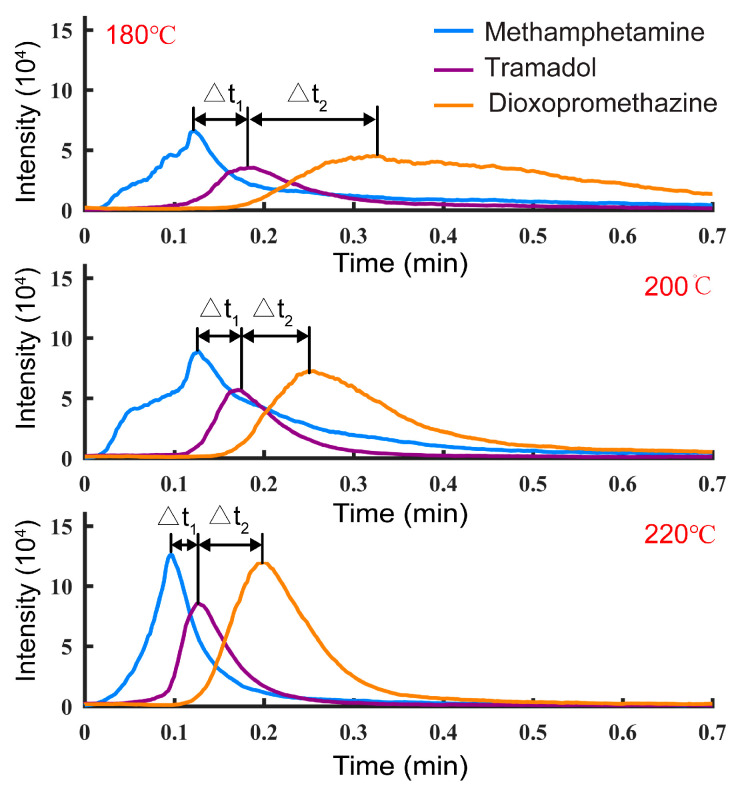
The extracted ion currents of methamphetamine, tramadol, and dioxpromazine hydrochloride in the mixed solutions at different TD temperatures.

In terms of TD, temperature is a key factor affecting the separation performance. To investigate the influence of temperature on the separation performance for mixed samples, a mixed standard solution of 1 μg/mL methamphetamine, tramadol, and dioxpromazine hydrochloride was prepared with methanol, and 10 μL samples were detected by TD-CDI at different temperatures. The change in extracted ion currents (EICs) is shown in Figure 5. The experimental results show that as the temperature increases, the intensity of the signal peak increases, the desorption time decreases, and the desorption time difference of different analytes decreases. On the other hand, at high temperatures, when the signal of a certain target is the strongest, the signals of other substances to be measured and the background noise will be relatively weak. Therefore, increasing the temperature can improve the desorption efficiency and achieve a better material separation effect. To achieve better detection performance, the desorption temperature was set at 240 °C in the following experiments.

### 3.3. Analyses of Drugs with Similar m/z

**Figure 6:**
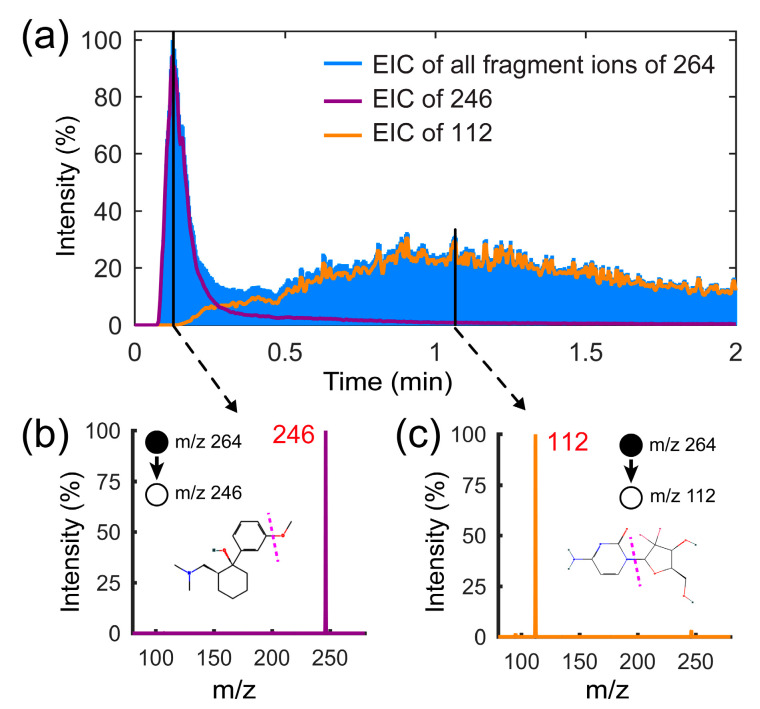
(**a**) The extracted ion currents of the fragment ions of tramadol and gemcitabine mixed solution. (**b**) The tandem mass spectrum of tramadol (*m*/*z* 246); (**c**)The tandem mass spectrum of gemcitabine (*m*/*z* 112).

Following the optimization of TD-CDI, the feasibility of separating and detecting drugs with the similar mass needs to be further verified. Mass spectrometry has gradually become the gold standard for detecting illegal drugs. Some criminals will disguise illegal drugs as some drugs of similar mass to evade MS detection. For some isomers, even tandem mass spectrometry struggles to distinguish them effectively. This is a major challenge for the control of illegal drugs. Depending on the desorption separation capability of the TD module, some analytes with similar mass but different boiling points can be effectively distinguished and identified. We configured a mixture of tramadol (CAS: #27203-92-5; exact mass: 263.18 g/mol; boiling point: 388.1 °C) and gemcitabine (CAS: #95058-81-4; exact mass: 263.07 g/mol; boiling point: 482.7 °C) with a concentration of 1 μg/mL using the methanol solvent. The test results obtained using the TD-CDI are shown in Figure 6. Since the two analytes have the same *m*/*z* of 264, the EIC results for all fragment ions can be obtained through collision-induced dissociation (CID). During the experiment, 10 μL of mixed solution was added to the TD room, with a desorption temperature of 240 °C, corona voltage of 5 kv, CID isolation width of 1 *m*/*z*, and collision energy of 30%. As shown in Figure 6a, two peaks are observed successively. The results of the tandem mass spectra in Figure 6b,c prove that tramadol, which has a low boiling point, is desorbed first, while gemcitabine is desorbed later. The experiment proves the high qualitative capability of the TD-CDI device.

### 3.4. Detection of Actual Samples

**Figure 7:**
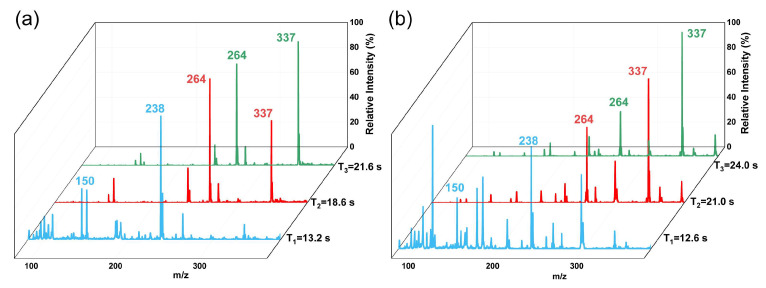
Spectra obtained by using TD-CDI to detect drugs in (**a**) milk tea and (**b**) urine samples.

The ability of TD-CDI to detect actual samples also needs to be further verified. Liquid drinks and powders are common drugs camouflage forms. Urine, saliva, blood, and hair testing are common samples for detecting drug abuse. In this study, the TD-CDI was used to detect drugs in capsules, milk tea, urine, and hair. Complex liquid beverages and human samples usually contain salt, protein, and other substances. In order to reduce interference from the complex matrix and achieve enrichment of target analytes to improve detection efficiency, samples were pre-treated by salting-out assisted liquid–liquid extraction (SALLE) before TD-CDI was used to detect milk tea and urine samples. MgSO_4_ is used as a salting-out agent, and acetonitrile is used as an extractant to obtain a good extraction performance. A 1 μg/mL mixed drug solution of methamphetamine (*m*/*z* 150), ketamine (*m*/*z* 238), tramadol (*m*/*z* 264), and fentanyl (*m*/*z* 337) was prepared with milk tea. During the experiment, 2 mL of the sample was mixed with 0.4 mL of acetonitrile and sufficient magnesium sulfate. The mixture was then shaken thoroughly to obtain a layered sample. The lower layer contained salt and sugar dissolved in water, the middle layer contained deposited proteins and pigments, and the upper organic liquid was enriched with the target substance. Then, transfer the supernatant and add 1% (*v*:*v*) 100 μg/mL formic acid solution to facilitate ionization. And take 10 μL of the treated supernatant for detection. And the detection results are shown in Figure 7a. The characteristic peaks of the four drugs are clearly displayed in the spectra. The signals of methamphetamine and ketamine reached the highest intensity at almost the same desorption time of 13.2 s. Tramadol and fentanyl were subsequently desorbed and detected, and it only took 21.6 s for all analytes to be desorbed. In the desorption time of each analyte, only the characteristic peaks of one or two drugs can be clearly observed, and the spectrum separation performance is good. Then, artificial urine was used as the solvent to prepare a 1 μg/mL mixed drug urine sample containing methamphetamine, ketamine, tramadol, and fentanyl. The mixed sample was subjected to pretreatment and detection under the same conditions above. The spectra obtained are shown in Figure 7b, and the characteristic peaks of the four drugs can be clearly observed, indicating good separation performance.

**Figure 8:**
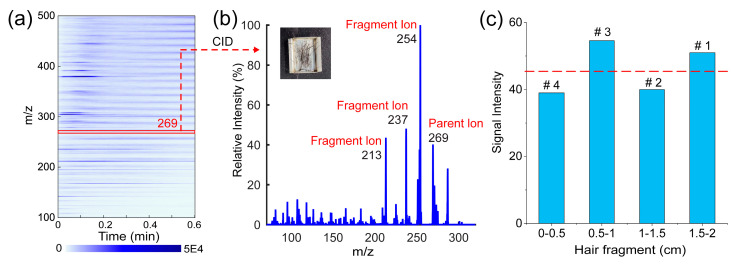
(**a**) The full scan ion map of the hair of the volunteer after taking Anweiyang capsules; (**b**) the tandem mass spectrum of formononetin in hair; and (**c**) the signal intensities for the characteristic fragment ion (*m*/*z* 254) of formononetin in different hair fragments (#1 represents ~0–10 days, #2 represents ~10–20 days, #3 represents ~20–30 days, and #4 represents ~30–40 days).

In order to further explore the ability of the TD-CDI to detect drugs in hair, a test experiment was conducted on the hair of a volunteer who followed the treatment courses of Anweiyang Capsules. The main ingredient of this capsule is 7-Hydroxy-3-(4-methoxyphenyl)-4H-chromen-4-one with the *m*/*z* of 269, also known as formononetin in flavonoids. We took a section of hair from the volunteer, washed it twice with acetone, put 5 mg directly into the injector, and used TD-CDI-MS for detection. As shown in Figure 8a, the full scan ion map of desorption substances in hair is very complex, which is why the general AIMS method is not effective in detecting hair. By utilizing the desorption separation capability of the TD module and the analytical capacity of CID tandem mass spectrometry, we were able to obtain the characteristic fragment ions information of formononetin in the hair of the volunteer after taking the drug for a period of time, as depicted in Figure 8b. It verifies the feasibility of using TD-CDI to detect drugs in hair.

The volunteer was treated with the drug for approximately two consecutive weeks (0–14 days), followed by a week (15–21 days) of drug withdrawal, followed by another two weeks (22–35 days) of drug treatment and then stopped taking the drug again (35–42 days). It is known that hair grows about 0.5 cm in 10 days. Hair was cut from the position close to the scalp of the volunteer and cut into sections every 0.5 cm from the hair root. Here, we assumed that drug levels would be similar at different hair sampling locations, and selected hair segments from the forehead and back of the head to form the test sample. A total of four sections were cut, and each section was ~5 mg. The signal intensity obtained by detecting each section of hair with the TD-CDI is shown in Figure 8c. The signals of formononetin detected in the 1.5–2 cm and 0.5-1 cm fragments were stronger, and the signals detected in other fragments were weaker. This result proves that the use of hair detection can determine whether the drug is used in the past period and the change in the dosage of the drug. These applied studies prove that TD-CDI has great potential in the field detection of drugs.

## 4. Conclusions

The escalating proliferation of illicit drug varieties has prompted widespread attention to diseases and illicit criminal activities associated with drug abuse. Traditional detection techniques, when applied to complex samples analysis, encounter challenges in simultaneously embodying simplicity, expeditiousness, analyte separation, and the capacity to detection diverse drug forms. In this study, a TD-CDI was developed for the detection of drugs in various dosage forms. The structural design of the TD-CDI is characterized by its simplicity and compactness, while demonstrating a satisfactory performance in rapidly separating analytes. Notably, a single spectrum can exhibit peaks corresponding to only one or two analytes, significantly enhancing the substance identification capability of samples. The influence of analytes, solvents, and temperature on the performance of detecting single-standard and mixed-standard samples was systematically evaluated, and optimizations were subsequently conducted. The integration of TD-CDI contributes to diminishing matrix interference. In conjunction with the LCQ-Fleet ion trap mass spectrometer, TD-CDI was applied to detect drugs in capsules, beverages, urine, and hair samples. By segmenting the analysis of drug content within hair samples, the possibility of tracing patients’ medication cycles is facilitated. The integration of TD-CDI and MS offers a satisfactory and versatile solution for the rapid and efficient detection of drugs within complex samples.

## Data Availability

The datasets generated during and/or analyzed during the current study are available from the corresponding author upon reasonable request.
